# Molecular Genetic Analyses of Polytene Chromosome Region 72A-D in *Drosophila melanogaster* Reveal a Gene Desert in 72D

**DOI:** 10.1371/journal.pone.0023509

**Published:** 2011-08-10

**Authors:** Monica T. Cooper, James A. Kennison

**Affiliations:** Program on Genomics of Differentiation, Eunice Kennedy Shriver National Institute of Child Health and Human Development, National Institutes of Health, Bethesda, Maryland, United States of America; University of Missouri, United States of America

## Abstract

We have investigated a region of ∼310 kb of genomic DNA within polytene chromosome subdivisions 72A to 72D of *Drosophila melanogaster*. This region includes 57 predicted protein-coding genes. Seventeen of these genes are in six clusters that appear to have arisen by tandem duplication. Within this region we found 23 complementation groups that are essential for zygotic viability, and we have identified the transcription units for 18 of the 23. We also found a 55 kb region in 72D that is nonessential. Flies deficient for this region are viable and fertile. Within this nonessential region are 48 DNA sequences of 12 to 33 base pairs that are completely conserved among 12 distantly related Drosophila species. These sequences do not have the evolutionary signature of conserved protein-coding DNA sequences, nor do they appear to encode microRNAs, however, the strong selection suggests functions in wild populations that are not apparent in laboratory cultures. This region resembles dispensable gene deserts previously characterized in the mouse genome.

## Introduction

The *Drosophila melanogaster* genome has been intensely studied for over 100 years. Recently, the sequencing of the majority of the genomic DNA has revealed much about the structure and organization of the genome [1]. In spite of the molecular advances, much still remains to be discovered about the functions encoded within the genome. Based on the results of characterizing small regions of the genome, it has been extrapolated that there are only about 3600 genes in Drosophila essential for viability [2]–[4]. Intensive efforts by the *Drosophila* Gene Disruption Project to mutagenize the genome with transposable element insertions have generated a collection of transposon insertions that tag about two-thirds of all annotated protein-coding genes [5], however, many of these transposon insertions do not affect the function of the tagged gene. While experiments to saturate small regions of the genome for mutations in essential genes are labor intensive, these experiments provide important genetic materials for understanding genome function. Therefore, we decided to identify and characterize the essential genes within a genomic region spanning about 22 polytene chromosome bands in subdivisions 72A to 72D of the third chromosome. This region includes 57 predicted protein-coding genes in 310 kb of genomic DNA. At least 23 of these genes appear to be essential for viability. We analyzed the transposon insertions in this genomic region from the *Drosophila* Gene Disruption Project to determine the level of saturation for gene function disruption among the tagged genes. In addition, we identified a large dispensable region reminiscent of gene deserts found in the mouse genome [6].

## Results

After EMS mutagenesis, we recovered 188 mutations that failed to complement *Df(3L)th102*. These mutations define 22 complementation groups. One mutant chromosome failed to complement mutations in two adjacent genes (*DNApol-delta^11^ Arf72A^3^*), and is probably a small deletion. The essential complementation groups and the number of alleles that we recovered for each are shown in Table 1. We recovered an average of 8.5 alleles per complementation group, with one complementation group [*l(3)72Ds*] represented by a single allele.

**Table 1:** Complementation groups represented by the mutations within the *Df(3L)th102* region of the genome.

Complementation Group	Number of Alleles	Identified Transcription Unit	Number of Core Amino Acids^a^	Number of Evolutionarily Conserved Amino Acids^b^	Proportion of Conserved Core Amino Acids
*l(3)72Aa*	19	*Brm*	1633	1231	75%
*l(3)72Ab*	17	*CG5931*	2142	2021	94%
*l(3)72Ac*	13^c^	*DNApol-delta*	1092	871	80%
*l(3)72Ad*	2	*Hip14*	637	561	88%
*l(3)72Ae*	3^c^	*Arf72A*	180	177	98%
*l(3)72Da*	8	*Notum*	671	349	52%
*l(3)72Db*	37	*mib1*	1226	948	77%
*l(3)72Dc*	11	*th*	438	164	37%
*l(3)72Dd*	11	*Mbs*	795	486	61%
*l(3)72De*	13				
*l(3)72Df*	9				
*l(3)72Dg*	10				
*l(3)72Dh*	2	*CG5161*	139	121	87%
*l(3)72Di*	3				
*l(3)72Dj*	4	*Taf4*	851	467	55%
*l(3)72Dk*	2	*Zn72D*	629	431	69%
*l(3)72Dl*	11	*Taspase1*	365	279	76%
*l(3)72Dn*	4	*CG5018*	696	206	30%
*l(3)72Do*	3	*CG34246*	240	70	29%
*l(3)72Dp*	4	*CG32155*	373	110	29%
*l(3)72Dr*	2	*CG32154*	345	35	10%
*l(3)72Ds*	1	*Med10*	133	120	90%
*l(3)72CDc*	0				

a Core amino acids are those present in all protein isoforms for that gene.

b Evolutionarily conserved amino acids are those core amino acids conserved among nine Drosophila species. Because of sequence gaps, we did not include in the analysis the *D. yakuba CG5931* gene or the *D. persimilis CG5931*, *DNApol-delta*, *th*, and *Mbs* genes. The *D. mojavensis CG32154* gene was not found by Blat, but was examined by BLAST to identify conserved aa.

c *DNApol-delta^11^* and *Arf72A^3^* were recovered on the same mutagenized chromosome.

We also tested mutations from other groups that were previously mapped to this region of the genome. The *kst^01318^* mutant chromosome was reported to carry a second-site lethal mutation, *l(3)72Dq^01318^*(http://flybase.org/reports/FBgn0028257.html), which failed to complement *Df(3)st-f13*. We could not confirm the existence of *l(3)72Dq^01318^*, as the *kst^01318^* mutant strain from the Bloomington Stock Center complemented both *Df(3L)st-f13* and *Df(3L)th102* for viability. Another complementation group that was mapped to this region is *E(smoDN)B-left* [7]. We found that *E(smoDN)B-left* is allelic to *l(3)72Dh*. Finally, Daniel Kalderon and co-workers screened for mutations that failed to complement *Df(3L)brm11*, and identified six complementation groups [*l(3)72CDa* through *l(3)72CDf*] that failed to complement both *Df(3L)brm* and *Df(3L)st-f13* [8]. We found that three of their complementation groups correspond to three of our complementation groups; *l(3)72CDa* corresponds to *l(3)72Db*, *l(3)72CDe* corresponds to *l(3)72Dc*, and *l(3)72CDf* corresponds to *l(3)72Da*. In addition, we confirmed the location of their complementation group *l(3)72CDc*, which is the 23rd essential gene within the region deleted by *Df(3L)th102*. We were unable to confirm their other two complementation groups. We found that *l(3)72CDb^M3^* complemented *Df(3L)th102*. We also found that the *l(3)72CDd* complementation group is an artifact. It is represented by a single mutant chromosome that failed to complement two deletions, *Df(3L)brm11* and *Df(3L)st-f13*. The *l(3)72CDd^L2^* mutant chromosome was assumed to carry a single lethal mutation in the region of overlap missing in both deletions [8], however, we found that it carries two different lethal mutations, one of which fails to complement each deletion. The lethality when heterozygous to *Df(3L)brm11* is caused by an *Arf72A* mutation, which we have named *Arf72A^L2^*. This is the only lethal mutation on this chromosome within *Df(3L)th102*, since the lethality over *Df(3L)th102* was rescued by the *Arf72A* transgene, *Arf72A^+t10.8^* [3]. The lethality when heterozygous to *Df(3L)st-f13*, is caused by a second mutation, *l(3)72-73a^L2^*, which also failed to complement *Df(3L)st-g24*.

**Figure 1:**
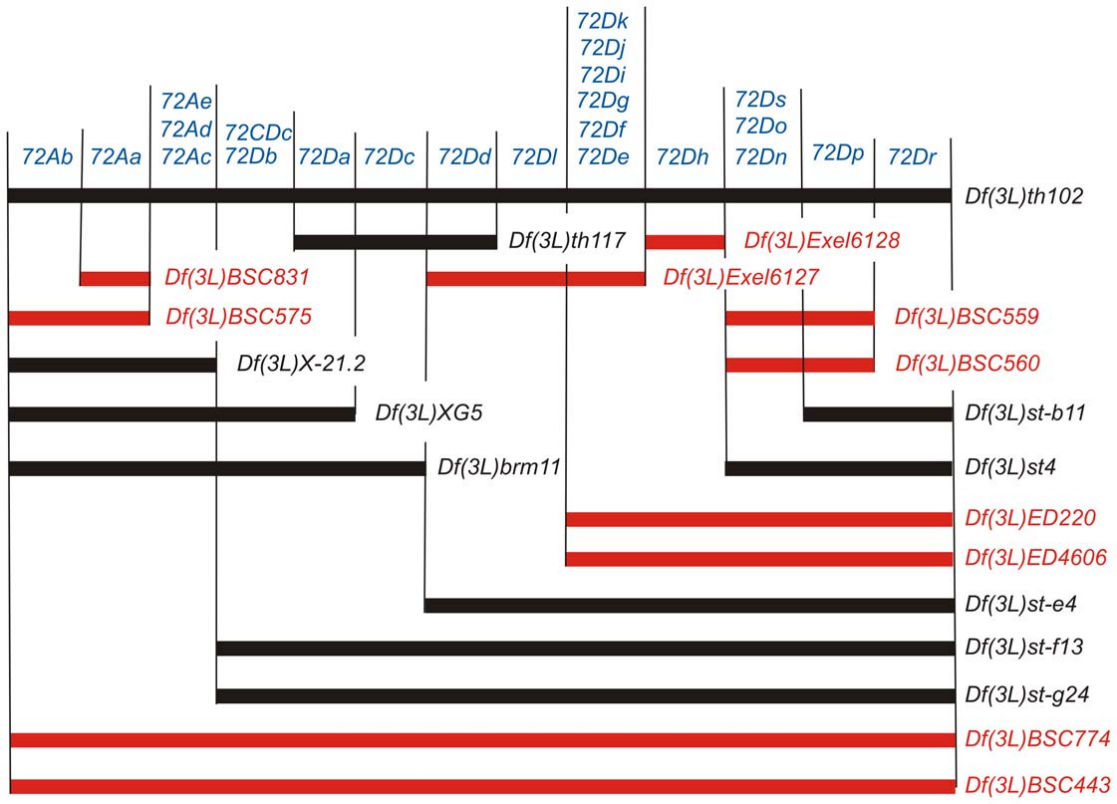
Complementation of essential genes with a set of deletions that overlap *Df(3L)th102*. The essential genes are listed at the top of the map in blue, with a horizontal bar for each deletion indicating which genes failed to complement that deletion. Deletions with molecularly characterized breakpoints are indicated by red bars. Deletions with no molecular information are indicated by black bars.

To further localize our complementation groups, we also crossed representatives of each complementation group to chromosomal deletions that overlap *Df(3L)th102* (shown in Figure 1). Ten of the deletions (those indicated by the red bars in Figure 1) have molecularly defined breakpoints, which were useful in integrating the genetic and molecular maps.

Nine of our complementation groups in Table 1 were previously correlated with the molecularly identified genes *brm*, *Arf72A*, *Hip14*, *Notum*, *mib1*, *th*, *Mbs*, *Taf4*, and *Zn72D* [9]–[17]. To identify the transcription units for our remaining complementation groups, we tested the putative lethal transposon insertion mutants in this region that the *Drosophila* Gene Disruption Project had made available from the Bloomington Drosophila Stock Center. These 20 transposon insertion mutants are listed in Table 2, and include P (P), piggyBac (PBac), and Minos (Mi) transposable element insertions. Nine of the transposon insertion mutants complemented *Df(3L)th102* for viability, indicating that the lethality of the insertion chromosome is not due to disruption of the associated gene. Eleven of the transposon insertion mutants failed to complement one of our complementation groups. The complementation groups that failed to complement each transposon insertion mutant are shown in Table 2. The locations from the deletion mapping coincided with the locations of the transposon insertions. We used this information to assign an additional five of our complementation groups to the molecularly identified genes shown in Table 1.

**Table 2:** Transposon insertion mutants identified by the *Drosophila* Gene Disruption Project and maintained by the Bloomington Drosophila Stock Center.

Transposon Insertion Mutants	Complementation Group Affected
*PBac{RB}CG5931^e03171^*	*l(3)72Ab*
*P{EPgy2}mib1^EY09780^*	*l(3)72Db*
*P{XP}Notum^d00939^*	*l(3)72Da*
*P{lacW}th^j5C8^*	*l(3)72Dc*
*P{EPgy2}th^EY20302^*	*l(3)72Dc*
*P{PZ}Mbs^03802^*	*l(3)72Dd*
*P{wHy}Taf4^DG08308^*	*l(3)72Dj*
*P{EP}CG5161^G4096^*	*l(3)72Dh*
*P{lacW}l(3)72Dn^j5A44^*	*l(3)72Dn*
*PBac{5PHw^+^}CG34246^B300^*	*l(3)72Do*
*P{EP}Med10^G18634^*	*l(3)72Ds*
*Mi{ET1}CG5027^MB04280^*	viable, but males and females poorly fertile
*P{EPg}HP36806*	viable and fertile
*P{EPgy2}Arf72A^EY03856^*	viable and fertile
*P{Mae-UAS.6.11}Pgm^LA00593^*	viable and fertile
*P{lacW}SsRbeta^s1939^*	viable and fertile
*P{EPgy2}CG32152^EY05944^ CG5151^EY05944^*	viable and fertile
*P{SUPor-P}CG5151^KG01027^*	viable and fertile
*P{SUPor-P}KG10105*	viable and fertile
*PBac{PB}PDCD-5^c04145^*	viable and fertile

We assigned three of the remaining complementation groups to transcription units by sequencing candidate genes (suggested by the deficiency mapping) from homozygous mutants. We sequenced the *CG32155* gene from two alleles of the *l(3)72Dp* complementation group. Both alleles were isolated on the *iso-1* third chromosome. The *l(3)72Dp^1^* mutation has seven base pairs deleted and three base pairs inserted (a net loss of four base pairs). This should frame shift the CG32155 protein after residue T32, causing the addition of six amino acid residues (VFTSMV) before a stop codon truncates the protein. The *l(3)72Dp^3^* mutation is a GC to AT transition that changes amino acid residue W128 to a stop codon, prematurely truncating the CG32155 protein. We sequenced the *CG32154* gene from two alleles of the *l(3)72Dr* complementation group. Both alleles were isolated on the *red^1^ e^4^* chromosome, which differs from the *iso-1* sequence at amino acid 206 (S in iso-1 and C in the *red^1^ e^4^* marked chromosome). Each allele has one additional amino acid change from the parental chromosome, both caused by TA to CG transitions. The *l(3)72Dr^1^* mutation changes amino acid residue C323 to R and the *l(3)72Dr^2^* mutation changes amino acid residue N258 to S. We sequenced the *Taspase1* gene from seven *l(3)72Dl* alleles. The *Taspase1* alleles *1*, *3*, *5*, *6*, *7*, *8*, and *9* change amino acid residues D75 to V, P98 to L, C74 to Y, E253 to K, G197 to E, P98 to L, and G252 to S, respectively. All of the *Taspase1* alleles died at the pharate adult stage when heterozygous to *Df(3L)th102*. Although Taspase1 cleaves the homeotic transcriptional regulator Trithorax [18], [19], we did not identify any homeotic defects in patterning of the adult cuticle. The late lethality of the *Taspase1* mutants is probably due to the perdurance of maternally encoded gene products. We tried to eliminate the maternally encoded gene products by making germ-line clones [20] with two of the stronger alleles, *Taspase1^6^* and *Taspase1^8^*, but the *Taspase1* mutant clones failed to produce mature eggs.

Finally, we used imprecise excision of the P element insertion *P{EP}DNApol-delta^EP3292^* to recover *DNApol-delta^14^*, which failed to complement *l(3)72Ac* alleles. Using all of the information above, we have been able to assign 18 of the 23 complementation groups to transcription units.

**Figure 2:**
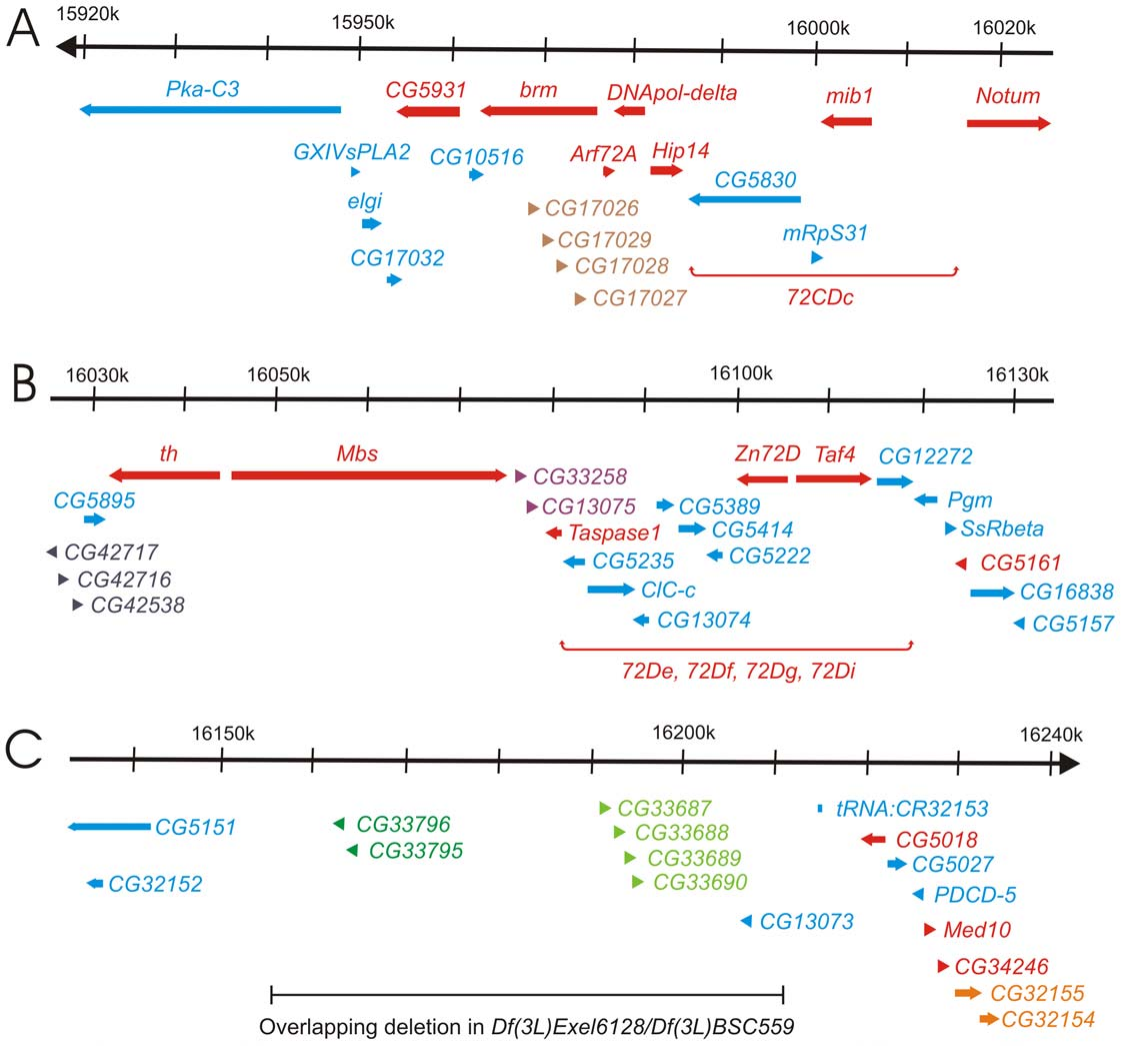
Molecular map of the genomic region deleted in *Df(3L)th102*. The approximately 320 kb of genomic DNA (from 3L: 15918k to 16240k, Release 5.23) is broken into three parts (A, B, and C), and is represented by the horizontal black arrows at the top of each part. The annotated transcription units are represented by colored thick horizontal arrows. The essential transcription units are red and orange. The clusters of transcription units encoding related proteins are brown (the cluster in 2A), grey (the cluster in 2B), purple (the cluster in 2B), dark green (the cluster in 2C), light green (the cluster in 2C), and orange (the essential genes *CG32155* and *CG32154* in 2C). All other transcription units are blue. The two regions that include the five essential genes (*72CDc*, *72De*, *72Df*, *72Dg*, and *72Di*) for which the transcription units have not been identified are indicated by red horizontal brackets below the candidate transcription units. The DNA missing in flies trans-heterozygous for the overlapping deletions *Df(3L)Exel6128* and *Df(3L)BSC559* is indicated by the horizontal black bar at the bottom of 2C.

We identified six clusters of genes in the 72A–D region of the genome that appear to have arisen by tandem gene duplication. The most distal cluster of related genes in 72A–D (the brown-colored transcription units *CG17026*, *CG17029*, *CG17028*, and *CG17027* in the top panel of Figure 2) is within a large intron of the *brahma* gene, and encodes putative inositol monophosphatases that are 41%–77% identical to each other. There are four genes in all Drosophila species except *D. willistoni* (three genes) and *D. yakuba* (eight genes). In the more distantly related dipteran, the mosquito *Anopheles gambiae*, there is only a single ortholog in the intron of the *brahma* gene. The next most distal cluster of three related genes (the grey-colored transcription units *CG42717*, *CG42716*, and *CG42538* at the left of panel B in Figure 2) encodes putative members of the BPTI/Kunitz family of serine protease inhibitors that are 32–43% identical to each other. There is a single gene in *D. mojavensis*, but between two and six genes in the other Drosophila species that we examined. We were unable to identify an ortholog in *A. gambiae*. The third cluster of related genes (*CG33258* and *CG13075*, the purple-colored transcription units between *Mbs* and *Taspase1* in Figure 2) encode putative chitin-binding proteins that are 49% identical to each other. There is only a single gene in *D. grimshawi*, but two genes in all other Drosophila species. However, the ortholog of *CG33258* in *D. persimilis* and *D. pseudoobscura* appears to have transposed and is now adjacent to *rdgC* (located at 77B1 about 4 Mbp proximal to 72D in *D. melanogaster*). We were unable to identify an ortholog for these proteins in *A. gambiae*. The most proximal cluster of tandemly related genes is a pair of genes (*CG32155* and *CG32154*, indicated by the orange-colored transcription units at the bottom right of Figure 2) that encode putative gamma-glutamyl hydrolases that are 35% identical to each other. There are two genes in all Drosophila species, but only a single ortholog in *A. gambiae*. Both *CG32155* and *CG32154* are essential for viability in *D. melanogaster*.

**Figure 3:**
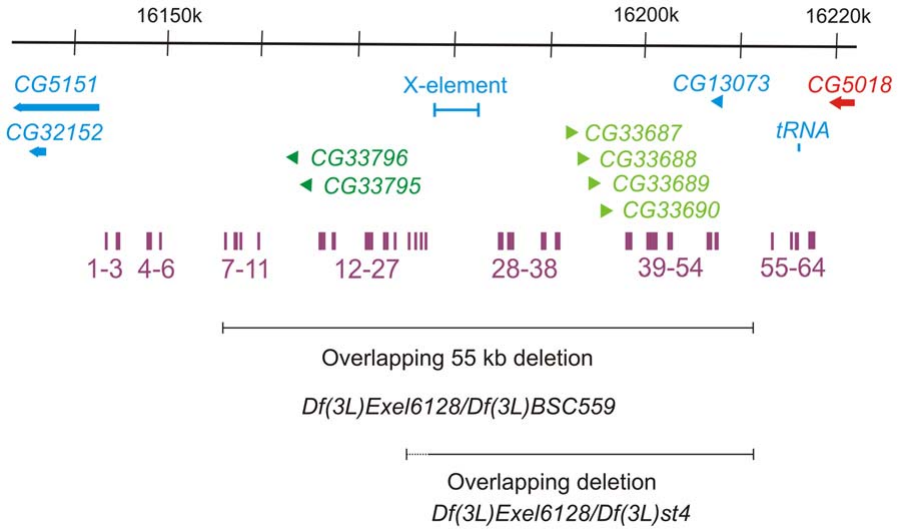
Molecular map of the ∼80 kb genomic region between *CG5151* and *CG5018*. The approximately 80 kb of genomic DNA (from 3L: 16140k to 16220k, Release 5.23) is represented by the horizontal black arrow at the top. The predicted transcription units are represented by colored thick horizontal arrows. The essential transcription unit *CG5018* is red. The clusters of transcription units predicted to encode related proteins are dark green and light green. All other transcription units are blue. The transposable X-element (shown by the blue bar) is present in the iso-1 strain, but not in Canton S. The highly conserved DNA sequences are represented by the purple bars in the middle. Below each cluster of conserved sequences are the numbers corresponding to Table S1. The DNA sequences missing in flies trans-heterozygous for the overlapping deletions *Df(3L)Exel6128* and *Df(3L)BSC559*, or *Df(3L)Exel6128* and *Df(3L)st4* are indicated by the horizontal black bars at the bottom. The dotted line at the left of the *Df(3L)Exel6128/Df(3L)st4* black bar indicates the 3kb region to which the distal breakpoint of *Df(3L)st4* was mapped.

Two additional clusters of related genes in 72D are shown in Figures 2 and 3. Each cluster appears to have originated by tandem duplication, with two predicted genes in the distal cluster (*CG33795* and *CG33796*) and four predicted genes in the proximal cluster (*CG33687*, *CG33688*, *CG33689*, and *CG33690*). In addition, the two clusters are distantly related to each other. In the *D. melanogaster iso-1* strain, there is an *X* element non-LTR transposon insertion between the two clusters. This *X* element insertion is not present in the *Canton S* strain (data not shown). No cDNAs have been isolated for any of the genes in either cluster, nor did any of them show expression in the high-throughput RNA sequencing (RNA-seq) data from the modENCODE project [21]. We generated flies that lack ∼55 kb of genomic DNA [*Df(3L)Exel6128/Df(3L)BSC559* transheterozygotes and *Df(3L)Exel6128/Df(3L)BSC560* transheterozygotes] that includes both clusters and another predicted gene, *CG13073* (Figures 2 and 3). We used PCR with multiple primers to verify that the DNA in this region was missing as expected (data not shown). The flies lacking this ∼55 kb showed no decrease in viability and were fertile. They had no discernable phenotypes when derived from heterozygous parents. However, when we inbred the deficient flies for several generations, their progeny often had thin bristles and etched abdominal tergites, characteristics of both *bobbed* mutations (mutants in the rDNA clusters on the X and Y chromosomes) and Minute mutations (genes encoding ribosomal proteins). These phenotypes only appeared after several generations of inbreeding and could not be rescued by two large paternally inherited duplications (*Dp(3;Y)ST1* and *Dp(3;Y)L131-D3*). We believe that the phenotype is not caused by deleting this region, but by a maternal-effect mutation somewhere else in the genome. Another overlapping pair of deletions, *Df(3L)Exel6128* and *Df(3L)st4*, delete about two-thirds of the same dispensable region (Figure 3). Inbreeding the *Df(3L)Exel6128/Df(3L)st4* transheterozygous flies for several generations did not reveal the same Minute-like phenotype as observed when inbreeding the *Df(3L)Exel6128/Df(3L)BSC559* or *Df(3L)Exel6128/Df(3L)BSC560* transheterozygous flies.

## Discussion

Six clusters of genes in the 72A–D region of the genome appear to have arisen by tandem gene duplication. These clusters include 17 of the 57 predicted protein-coding genes. In a previous study of the 76B–D region, we identified four clusters of related genes, which included 17 of the 80 protein-coding genes (excluding the polymorphic *Gyc76C* duplication in the *iso-1* strain) [22]. In the Adh region, it was reported that at least 38 of the 218 predicted protein-coding genes are in clusters that appear to have arisen by tandem duplication [23]. For all of these regions, the frequencies of related gene clusters appear to be significantly higher than the frequency first reported for the entire genome [24]. The presence of large numbers of tandem gene duplications may help to partially explain the finding that the estimates of the total number of essential genes determined by mutational analyses [2]–[4] are significantly less than the number of genes found by molecular analyses [1]. Until duplicated gene pairs have diverged sufficiently to have some non-overlapping functions, both genes must be mutated simultaneously to cause a mutant phenotype. The more recent the gene duplication event, the more likely it is that the duplicated gene pair will be refractory to mutational analyses.

Over the past 20 years, the *Drosophila* Gene Disruption Project has screened >200,000 independent transpositions to assemble a collection of transposon insertions that tag about two-thirds of the annotated protein-coding genes [5]. They did not determine the proportion of the tagged genes that were functionally disrupted by the transposon insertions. Therefore, we used the 47 essential genes identified here and in our previous characterization of the 76B–D region [22] to estimate this proportion. We found that 21 of the 47 essential genes (45%) were functionally disrupted by the *Drosophila* Gene Disruption Project collection.

What proportion of mutations decrease gene function enough to cause a mutant phenotype? We can estimate this proportion from the data reported here and in our previous work [22]. We identified the transcription units for 37 of the essential genes in the two chromosomal regions that we characterized. There are 31338 core amino acid residues (or 9.4×10^4^ base pairs) in these 37 essential genes. Thus, each mutagenized third chromosome that we screened with both deletions was 9.4×10^4^ base pairs of open reading frame tested. We did not screen all of our mutagenized chromosomes with both deletions. Therefore, we have confined our analysis to the 3009 EMS-treated third chromosome lines that we screened with both chromosomal deletions. We thus tested a total of 2.8×10^8^ base pairs (9.4×10^4^ base pairs on each of the 3009 chromosomes) for lethal mutations in our 37 identified essential genes. We recovered 130 mutations, which corresponds to one mutation in every 2.2×10^6^ base pairs (2.8×10^8^ total base pairs tested divided by 130 mutations recovered), or one mutation per 2200 kb. This is the estimate for mutations that cause a lethal mutant phenotype. We can compare this to the estimates for the frequency of mutations that alter DNA sequence (but may not necessarily cause a mutant phenotypes). The latter frequencies range from 1 mutation per 273 kb to 1 mutation per 476 kb [25], [26]. Thus, the frequency of base pair changes in the DNA after EMS treatment is 5 to 8 times the frequency of mutations that actually affect gene function sufficiently to cause a mutant phenotype.

The region between *CG5151* and *CG5018* in 72D9–10 (a region of ∼78 kb) may be similar to gene deserts that have been described in mammalian genomes [6], [27]. While there are seven predicted genes, there is only experimental evidence for one of these (*CG13073*). At least 55 kb are dispensable. Two gene deserts in the mouse genome were deleted and were also dispensable [6]. We can identify this possible gene desert in other species of *Drosophila* using the flanking *CG5151* and *CG5018* genes, which are evolutionarily conserved. The region varies in size from slightly less than 60 kb in *D. yakuba* to almost 78 kb in *D. simulans* and *D. melanogaster*. No protein-coding genes are conserved among all species. We identified the ortholog of *CG13073* in this region in all species except *D. persimilis*. In the subgenus Drosophila, *CG11196* is present in this region, while in the subgenus Sophophora, *CG11196* is located between *Nup44A* and *Hey* on Muller element C (44A2 in *D. melanogaster*). We do not know which location for *CG11196* is the ancestral, as the *A. gambiae CG11196*, *Nup44A*, and *Hey* orthologs are in three different locations. While there are few, if any, genes conserved between *CG5151* and *CG5018*, there are many DNA sequences conserved. We identified 64 DNA sequences between 12 and 43 base pairs in length that are completely conserved among 12 *Drosophila* species. Forty-eight of the conserved sequences (7 through 54) are within the region deleted by both *Df(3L)BSC559* and *Df(3L)Exel6128*. The conserved sequences are in Table S1 and their approximate locations are shown in Figure 3. These sequences are not distributed randomly throughout the 78 kb region of *D. melanogaster*, but are clustered. For example, several pairs of sequences are separated by only a single variant nucleotide. Sequences 13 and 14 have 55/56 base pairs conserved, sequences 20 and 21 have 46/47 base pairs conserved, sequences 30 and 31 have 43/44 base pairs conserved, sequences 32 and 33 have 56/57 base pairs conserved, and sequences 45 and 46 have 51/52 base pairs conserved. These sequences do not have the evolutionary signatures of conserved protein-coding DNA sequences or of microRNAs [28]. We believe that they are probably target sites for DNA-binding proteins. Large numbers of evolutionarily conserved DNA sequences are also present in gene deserts in the mouse genome [6]. While many of these sequences are dispensable in the lab in both the mouse and in *D. melanogaster*, their strong evolutionary conservation suggests functions critical in nature.

## Materials and Methods

Flies were raised on a yeast/cornmeal/molasses/Tegosept medium at 25°. All mutations and chromosome aberrations are described in Lindsley and Zimm [29] or Flybase (http://flybase.org/) unless otherwise noted. For the *Taspase1* germ-line clones, three independent recombinants with *P{FRT(w^hs^)}2A* were tested for each allele using *P{ovoD1-18}3L P{FRT(w^hs^)}2A* as described [20]. We recovered *DNApol-delta^14^* as a derivative of *P{EP}DNApol-delta^EP3292^*. Putative deletions were initially detected by a change in eye color after crossing to the balancer *TMS* (which carries a transposon that expresses the P transposase), and were then tested for lethality when heterozygous to *Df(3L)th102*.

Males were fed ethyl methanesulfonate (EMS) as described [30], [31]. The mutagenized males were mated to virgin females and discarded after four days; the inseminated females were returned to new cultures for subsequent brooding. Mutagenized males were homozygous for either an unmarked chromosome from the *iso-1* strain [3], or a third chromosome carrying *red^1^* and *e^4^*. Both third chromosomes were made isogenic prior to mutagenesis. We recovered mutations that failed to complement *Df(3L)th102* from two different experiments. Following the nomenclature suggested by Lindsley and Zimm [29], we provisionally named the complementation groups *l(3)72Aa* through *l(3)72Ae* for those complementation groups distal to *Df(3L)st-f13*, and *l(3)72Da* through *l(3)72Ds* (excluding *l(3)72Dq*) for those complementation groups within *Df(3L)st-g24*. We did not use the name *l(3)72Dq* for any of our complementation groups, since that name was already in use (http://flybase.org/reports/FBgn0028257.html). The first experiment to recover mutations that failed to complement *Df(3L)th102* was previously described [3]. In that experiment, we recovered 102 mutations that failed to complement *Df(3L)th102*; 26 of those mutations that failed to complement *Df(3L)brm11* (but complemented *Df(3L)st-f13*) were previously reported [3]. Two of the mutations from this experiment that failed to complement *Df(3L)th102* are alleles of *l(3)72Dm* [29]. We did not include *l(3)72Dm* or its alleles in our description of the genes within the 72A–D region, because the *l(3)72Dm* mutations complemented deletions that overlap *Df(3)th102*, and are probably allelic to a second-site mutation on the *Df(3L)th102* chromosome. In the second experiment, we generated balanced lines [22] with an EMS-treated third chromosome carrying the mutations *red^1^* and *e^4^*. Only those lines in which few or no flies homozygous for the mutagenized third chromosome survived were subsequently tested by crossing to *Df(3L)th102/TM6B* virgins. We tested 1938 lethal-bearing third chromosome lines and recovered 88 mutations that failed to complement *Df(3L)th102*.

We used inverse PCR [32] to determine the locations of transposon insertions. The approximate location of the distal breakpoint of *Df(3L)st4* was determined by PCR amplification of DNA fragments from *Df(3L)Exel6128/Df(3L)st4* transheterozygous flies. The distal breakpoint of *Df(3L)st4* is proximal to the DNA sequence CCGTTACACGTTGTACACC (base pairs 16173092 to 16173111 of the third chromosome, Release 5.30) and distal to the sequence CGAGGAGTTAAGGTCTCAG (base pairs 16176676 to 16176694 of the third chromosome, Release 5.30).

For the evolutionary comparisons, we used both BLAT on the UCSC Genome Browser website (http://genome.ucsc.edu/) [33] and BLAST on the NCBI website (http://blast.ncbi.nlm.nih.gov/Blast.cgi). For the determination of evolutionarily conserved amino acids, we used EvoPrinter Version 1.1 (http://evoprinter.ninds.nih.gov/index11.html) [34]. Since EvoPrinter Version 1.1 will only compare a maximum of nine species, we used D. melanogaster and the eight most distantly-related Drosophila species (*D. erecta*, *D. yakuba*, *D. ananassae*, *D. pseudoobscura*, *D. persimilis*, *D. virilis*, *D. mojavensis*, and *D. grimshawi*) with available BLAT files. Only those amino acid residues that were identical in all nine Drosophila species were counted as evolutionarily conserved. For the determination of evolutionarily conserved base pairs, we used EvoPrinterHD (http://evoprinter.ninds.nih.gov/evoprintprogramHD/evphd.html) [35].

## Supporting Information

Table S1 **Sequences (12 base pairs or longer) from the 72D region that are conserved among Drosophila species.**
(DOC)
