# Supplementary material for: Molecular Genetic Analyses of Polytene Chromosome Region 72A-D in Drosophila melanogaster Reveal a Gene Desert in 72D
Source: PLoS One. 2011 Aug 10;6(8):e23509. doi: 10.1371/journal.pone.0023509 (PMC3154481; doi:10.1371/journal.pone.0023509)
Supplement: Table S1 — Sequences (12 base pairs or longer) from the 72D region that are conserved among Drosophila species. (DOC) [file pone.0023509.s001.doc]

| Name | DNA sequence |
| --- | --- |
| 1 | GATAGCAGACGACTTAAG |
| 2 | GCACTTTAGACTTTTGCGCATTCCCAGCC |
| 3 | AGGTGACTCAAC |
| 4 | GCAACATTGTGTCATA |
| 5 | AGAGGCTTCGAAATATTAC |
| 6 | CAAACAAAATGATTAG |
| 7 | ACGGAAGTGATTTGCCTA |
| 8 | TTATGACAGCCACAAAAA |
| 9 | AATTTATTGCATTT |
| 10 | TTGACAGGAAGTCGCCGG |
| 11 | TTTATTTATGCAGCTTT |
| 12 | AAAGAATCTATTAAGAT |
| 13 | GTAGACATTTTTCGTATGACAACG |
| 14 | CAAATATTTGCCGGATAATATGAATAACAAA |
| 15 | TGTCGCCGGTGTCT |
| 16 | GCAGACAACTTCA |
| 17 | AAGGATACACAAGT |
| 18 | TCTCATTAAAAGAAA |
| 19 | GCAAAGTGGCTCAATGTCAGAC |
| 20 | TGAAACAAAATACCGAAATTCTA |
| 21 | GCGTGACCTTGAAAAGTATTCCT |
| 22 | TCGGTGGTTGTTTGT |
| 23 | TGCATTTTGACGTCAG |
| 24 | AAACGCATGAAATG |
| 25 | TGTCTGCACCGGCGTCT |
| 26 | AAGTGAAGCGTAAGCTGAAATGCACTCCCACTC |
| 27 | TTAACCTTGAAAAACGAAC |
| 28 | GCTTTTTATTTGACTTAT |
| 29 | TCGCTTAACTTTTT |
| 30 | GCGCAAACATTTGC |
| 31 | AGTATTAATTAACGCTTTGTTGAAGTTTG |
| 32 | CACTCAATTATTCAGGCATAATAAG |
| 33 | AAATTGTTTTAATATGCATAAATATCTTGTA |
| 34 | AGTCAAAACTGCCAGACAA |
| 35 | CGATCAATGCACCTT |
| 36 | CAAAGGTGCAACG |
| 37 | GCAGACAAATTTATGAG |
| 38 | TAAGTGAAGGCCAATAAAATT |
| 39 | TGCATTAAAATGTTTGTAATTGCA |
| 40 | CCATAGATGCCGCC |
| 41 | GTAACTATAATTGCCATAATAACATAATATC |
| 42 | CTTGTTTGTCAAA |
| 43 | AAACAGTTAAATGCCA |
| 44 | TCGGAATCAAAGCGAAAACAATGACACA |
| 45 | AAAAAAAATCGTCTAGACA |
| 46 | AGACTAGACTCCATAAAAATAATTATTATCGG |
| 47 | TAATTGCCACAATTTGCT |
| 48 | TGTCATATTTGAGGTG |
| 49 | GCCATAAATCAACTGC |
| 50 | CTGCAACAATGTAG |
| 51 | ATTATTTGCACTAT |
| 52 | GGCACCGCGAGGGC |
| 53 | GGCCCCCGCGGCGCC |
| 54 | TGGGGGCCACAGCGTC |
| 55 | CATATTGATTGAAATTT |
| 56 | AAATGCAAATCACTTAAA |
| 57 | CACGTTTGTCCTTA |
| 58 | CAACCGCATGCGATTTATGC |
| 59 | TTATTAAATGCAAATT |
| 60 | GGCTAATTTGCAAACCAATTAAATGTCAATTTGAGGCATTGTT |
| 61 | CAATGAGTTTTT |
| 62 | CTAAAAGTTGAATTGTGCA |
| 63 | TGTCTGTGTGGTATGCA |
| 64 | TGGCTAGACAGTGTAAAACCAA |
